# Awareness and Satisfaction About COVAXIN Vaccination Services at an Immunization Clinic in Nagpur: A Cross-Sectional Study

**DOI:** 10.7759/cureus.20983

**Published:** 2022-01-06

**Authors:** Ujwala U Ukey, Sanjeev M Chaudhary, Sarita K Sharma, Uday W Narlawar, Ravikant Singh, Aditi J Dabir

**Affiliations:** 1 Department of Community Medicine, Government Medical College & Hospital, Nagpur, IND; 2 Department of Community Medicine, Government Medical College & Hospital, Akola, IND

**Keywords:** cross-sectional, awareness, covaxin, immunization, elderly population, satisfaction

## Abstract

Introduction

Initially, coronavirus disease 2019 (COVID-19) vaccination was started in India for the elderly above 60 years of age. Adults with any comorbidity have been gradually included in the vaccination drive. It is empirical to gain insight into the satisfaction of these beneficiaries with the vaccination as it may act as an influencing factor for receiving the vaccine.

Materials and methods

This was a descriptive cross-sectional study carried out at the COVID-19 vaccination clinic of the Government Medical College and Hospital, Nagpur, among individuals above 60 years of age and those from 45 to 60 years of age with comorbidity. The survey tool was a predesigned structured questionnaire that had close-ended questions on various aspects of awareness about the COVID-19 vaccines and their satisfaction with the immunization center. Interviews were conducted by two interviewers on each day. Data were analyzed using open software Epi Info (CDC, Atlanta, Georgia). The chi-square test was applied as a test of significance.

Results

A total of 290 subjects participated in the study. The majority had correct knowledge about COVID-19 vaccination and appropriate COVID-19 behavior after vaccination. Fever and body ache were known to most of the subjects as adverse effects following immunization. Social media was the most common source of knowledge. The majority of the subjects were satisfied with the services provided at the vaccination center, but there was no difference as per age, gender, or residential status of the subjects.

Conclusion

Despite mixed rumors about the COVID-19 vaccine, the majority of the study subjects were well satisfied with the vaccination. They were apparently having fair awareness about the vaccine.

## Introduction

Pandemics have ravaged the human race since time immemorial [1], nevertheless, the current pandemic differs from the previous ones. As with other previous pandemics, this one is also related to emotions of enormous fright, anxiety, and botherations [2,3]. This pandemic is distinctive in terms that people are worried not only about getting infected or transmitting the disease to others but also have suffered societal and economic concerns due to the measures that were undertaken by the governments to curb the disease [2], such as home quarantine, the practice of social distancing, and nationwide lockdown and curfews, which have also led to several changes in day-to-day activities, staying indoors for increased length of time, travel restriction, limited access to essential needs, prolonged separation from families, and loss of jobs [4,5]. The current pandemic has thus resulted in havoc by affecting almost all the continents globally causing 281,808,270 cases with 5,411,749 deaths [6], endangering health, economy, life in all the ways, and international consonance, along with the spread of misinformation and panic in the world [7]. In India, the second most affected country of the world, 11,787,534 confirmed coronavirus disease 2019 (COVID-19) cases, and over 160,726 deaths were reported till 25 March 2021 [1] before the occurrence of the second wave. As of 29 December 2021, India has reported a total of 34,808,886 cases of COVID-19 and 480,592 deaths [8].

Although non-pharmacological interventions (NPIs) were able to slow down the progression of the disease [2,4], vaccination is perceived as a key strategy for halting the escalation of the COVID-19 pandemic [9]. The Government of India has introduced vaccination against COVID-19 in India from January 2021 to curtail the problem of the ever-burgeoning cases and to introduce herd immunity. The initial phase of the COVID-19 vaccine included the inoculation of healthcare workers and front-line workers and was further extended to individuals above 60 years of age and those above 45 years with comorbidities during the second phase. In Maharashtra, COVAXIN (Bharat Biotech, Hyderabad, India), the Indian brand, is being administered in four cities including Nagpur. COVAXIN is a whole virion, inactivated coronavirus (severe acute respiratory syndrome coronavirus 2) vaccine, which was developed in India by Bharat Biotech International Limited in partnership with the National Institute of Virology (NIV) and the Indian Council of Medical Research (ICMR). The Central Drugs Standard Control Organisation (CDSCO) approved the use of COVAXIN in India on 3 January 2021 [10]. Only minor side effects such as body ache, headache, fever, local injection site pain, redness, or swelling have been reported about it.

Various studies carried out the world over have proved the effectiveness of vaccination in decreasing morbidity and mortality due to COVID-19 [11-13]. However, there is an evident uncertainty clouding the COVID-19 vaccines [2]. Focused efforts are not only essential to protect human rights but to ensure the effectiveness of the vaccination campaign [14,15]. Vaccination distribution plans need to ensure full accessibility for persons with disabilities and the elderly [16]. All such efforts and special provisions help to improve the satisfaction of the general population about a healthcare facility, which eventually affects the utilization of health services like immunization. With this background, the present study was conducted with the objectives to estimate the awareness of the population about the COVID-19 vaccine (COVAXIN in the present setting) and also to explore their satisfaction with vaccination services by a rapid assessment form.

## Materials and methods

Study design and setting

A descriptive cross-sectional study was carried out at the COVID-19 vaccination clinic of the Government Medical College and Hospital, Nagpur. It is a tertiary care hospital that is geographically located in central India on the eastern border of the state of Maharashtra and is easily accessible to three other neighboring states (Chhattisgarh, Madhya Pradesh, and Telangana). Thus, the institute caters to the health needs of individuals from a diverse socio-demographic profile. It is a vaccination center that is authorized by the local health authorities, namely, the Nagpur Municipal Corporation (NMC). This health facility was the only center in the northeast part of the Maharashtra state where COVAXIN was being administered when this study was conducted.

Study population

For the present survey, the people who came for getting themselves vaccinated against COVID-19 constituted the study participants. As the study was carried out during the phase of the immunization wherein individuals above 60 years of age (and those from 45 to 60 years of age with comorbidities) were immunized, as per the operational guidelines drafted by the Government of India and issued to the states, in this phase of the campaign, vaccination was provided only to elderly individuals and adults above 45 years of age with comorbidities. Hence, they were also included in the study along with the people above 60 years of age.

Data collection

It was a routine practice to observe the COVID-19 vaccine beneficiaries for 30 minutes after they receive the vaccine dose. Data collection was done during this 30 minutes observation period with the help of a face-to-face interview technique. It took around 10-15 minutes to ask all the questions in the survey tool and thus sufficient time was given for each interview. These interviews were conducted in the local vernacular languages in the study area, which were Marathi and Hindi. The participants were interviewed while they were being observed for the development of any immediate side effects in the waiting room.

Study tool

The survey tool was a predesigned structured interview schedule that had close-ended questions on various aspects of awareness about the COVID-19 vaccines, such as the number of doses, the route of administration, and adverse events, and the satisfaction of the vaccine beneficiaries with the vaccination services at the immunization center. The interview schedule had three sections; the first section dealt with general information about the study participants, the next section dealt with awareness about the COVID-19 vaccine based on the frequently asked questions (FAQs) from the Centers for Disease Control and Prevention (CDC) [17] and information on the COVID-19 vaccine by the World Health Organization [18], and the third section assessed the satisfaction with the COVID-19 vaccination services at the immunization center with the aid of the Rapid Assessment System (RAS) [19]. The interview schedule thus developed was reviewed by an expert panel for content validity and reliability. A pilot study was conducted on 15 study subjects for pretesting and assessing the feasibility. Based on the findings of the pilot survey, necessary changes were made to the interview schedule.

Sample size and sampling technique

According to a survey by the Government of India, about 97% of people were satisfied with the COVID-19 vaccination experience [20]. Based on this and taking the absolute precision as 2% with confidence level as 95%, the estimated minimum sample size was found to be 279. The survey was carried out from 13 March 2021 to 23 March 2021 on all the working days when the immunization clinic was open. The convenience sampling method was applied to achieve the sample size wherein daily 31 eligible subjects selected from all the people who had received the vaccine at the immunization center on that day were interviewed. The people who came for receiving a COVAXIN injection in the center were allotted a number between one and 100 (separately for the morning, afternoon, and evening sessions) to avoid any chaos during the vaccination process. With the help of a random number table, the researchers selected 10, 10, and 11 subjects from the morning, afternoon, and evening sessions, respectively, on all the survey days. Interviews were conducted by two interviewers on each day.

Ethical considerations

Ethical clearance was obtained from the Institutional Ethics Committee vide letter number 2325/EC/Pharmac/GMC/Nagpur. Informed consent was obtained from the study participants for their participation in the study after apprising them of the nature and purpose of the study. The participants were assured that their identity will not be revealed and the data collected from them will be used only for the research purpose.

Statistical analysis

Data obtained from the interviews were primarily entered in the printed interview schedules. The filled-in printed schedules were allotted a unique participant number, checked for completeness, and missing entries, if any, were addressed preferably on the same day. These data from all the interviews were further entered into Microsoft Excel spreadsheets (Microsoft Corporation, Redmond, WA) after coding. This database was prepared for each day on a separate sheet, which was labeled with the date on which data were collected. All the databases were merged when the entries of all the 290 participants were completed. Before the actual analysis, the data were also checked for any duplicate entries as well as for errors in data entry due to wrong codes and missing values. These data were then analyzed using Epi Info software version 7.2.5.0 (CDC, Atlanta, Georgia) [21]. For continuous variables, descriptive measures such as mean and standard deviation (SD) values were calculated. Frequency distribution was done and percentages were calculated for categorical variables. The chi-square test was applied as a test of significance. P-values less than 0.05 were considered statistically significant.

## Results

Socio-demographic details of the respondents

In the present survey on awareness of COVID-19 vaccine and satisfaction about services at immunization center, the minimum sample size to be achieved was 279. The total number of beneficiaries who participated was 290. The mean age of the study participants was 64.02 years with a standard deviation (SD) of 7.28 years. The minimum reported age of the study participants was 45 years and the maximum age was 90 years. Individuals in the age group of ≥60 to ≤69 years constituted the majority (193, 66.55%) of the study participants, and those in the age group of 45 to ≤59 were only 36 (12.41%). Male respondents were 146 (50.34%) and the remaining 144 (49.66%) were females. The other characteristics of socio-demographic information of the study participants are shown in Table 1.

**Table 1:** Socio-demographic information of study participants (n = 290).

Characteristics	Number	Percentage
Age in years		
<50	9	3.1
50 to 59	27	9.31
60 to 69	193	66.55
70 to 79	53	18.28
80 to 90	8	2.76
Area of residence		
Urban	251	86.55
Rural	39	13.45
Per capita monthly income in INR		
<25,000 (335 USD)	113	38.97
>25,000 (335 USD)	177	61.03
Any comorbidities		
Yes	149	51.38
No	141	48.62

Details on the history of COVID-19

In the present study, 183 (63.10%) respondents reported not having been diagnosed with COVID-19 while the remaining 107 (36.90%) gave a positive previous history of COVID-19. Similarly, the previous history of coronavirus infection in their family members was given by 127 (43.79%) participants whereas the remainder 163 (56.21%) mentioned that none of their family members were ever infected with the coronavirus.

Awareness regarding COVID-19 appropriate behavior

The observations regarding awareness of COVID-19 appropriate behavior after getting vaccinated are represented in Table 2.

**Table 2:** Awareness of the respondents about post-vaccination COVID-19 appropriate behavior (n = 290).

Statement	Agree, number (percentage)	Not sure, number (percentage)	Disagree, number (percentage)
It gives lifelong immunity	119 (41)	109 (38)	62 (21)
No need to wear a mask as you have taken the vaccine	53 (18)	21 (7)	216 (75)
No need to wash or sanitize hands as you have taken the vaccine	51 (18)	18 (6)	221 (76)

COVID-19 vaccine-related awareness

Awareness of post-vaccination COVID-19 appropriate behavior was fairly good among the study subjects. Around 75% of subjects disagreed with the statements like there is no need to wear a mask or no need to wash hands after getting vaccinated. The majority of the subjects were aware of minor details of the COVID-19 vaccine, such as its mode of administration and doses (Table 3).

**Table 3:** Awareness about some aspects of the COVID-19 vaccine (n = 290).

Statement about COVID-19 vaccine	Correct response, number (percentage)	Incorrect response, number (Percentage)
Mode of administration	272 (94)	18 (6)
Number of doses	248 (86)	42 (14)
Type of vaccines against COVID-19	193 (67)	67 (33)

**Figure 1:**
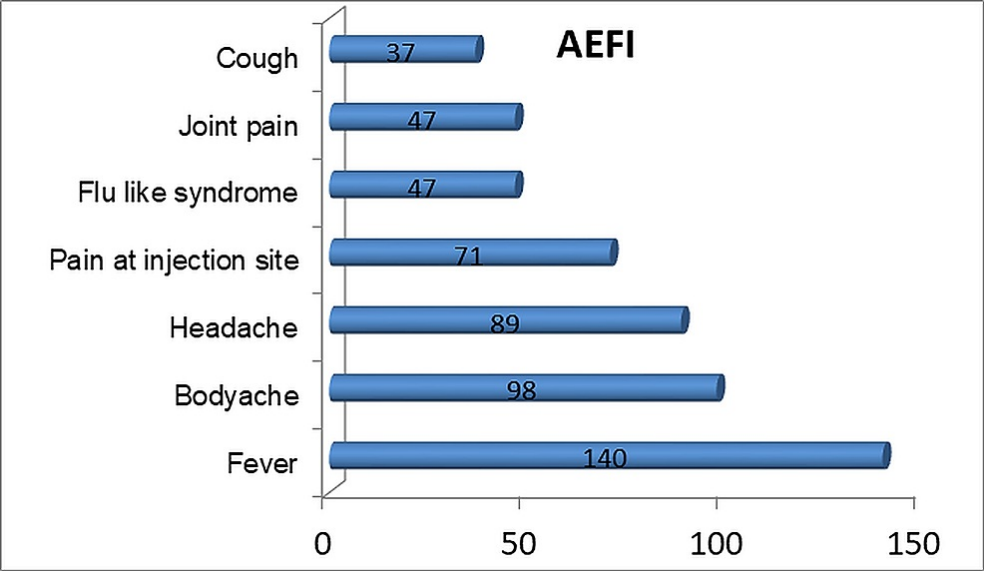
Awareness about adverse effects of COVAXIN. AEFI, adverse events following immunization.

Similarly, awareness about the side effects of COVAXIN was also good among the study subjects. Almost 48% (140 of 190) knew that fever was the most common side effect (Figure 1).

**Figure 2:**
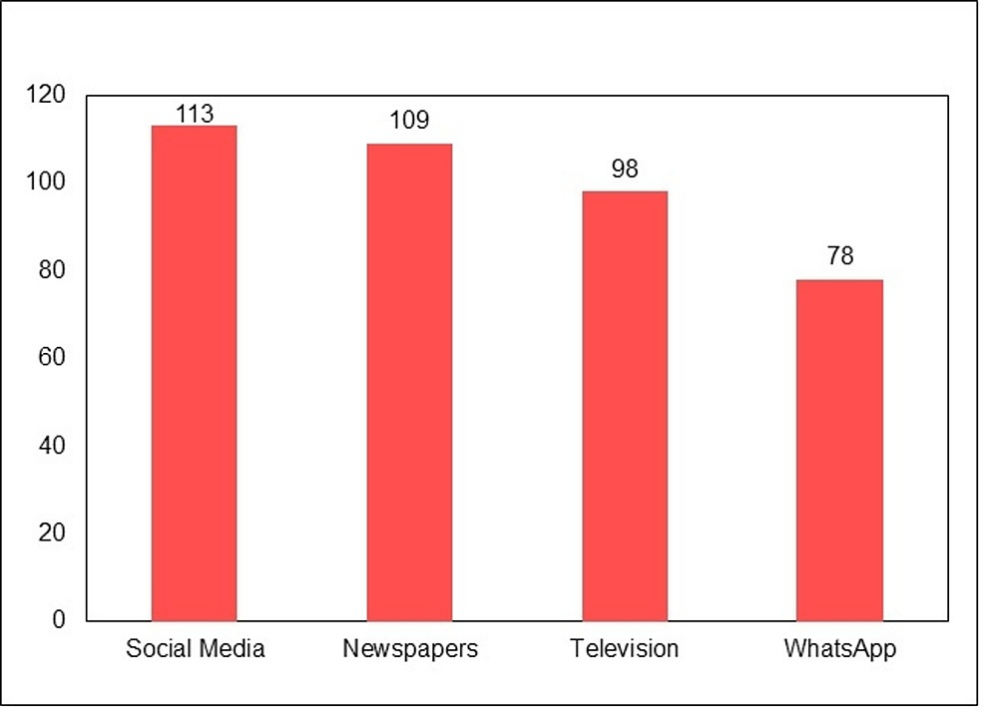
Source of information about COVID-19 vaccine.

Thus, the respondents had awareness about various adverse events such as fever, headache, body ache, and cough following the administration of the vaccine against COVID-19. The study participants were asked about the source of their knowledge on the COVID-19 vaccine. Social media (n = 113, 38.97%), television (n = 109, 37.59%), newspaper (n = 98, 33.79%), and WhatsApp (n = 78, 26.90%) were the sources of information as cited by the study subjects. Details regarding the same are displayed in Figure 2.

Satisfaction about COVAXIN

The vaccine beneficiaries were enquired about their satisfaction regarding the COVAXIN vaccination services at the center. The five-question RAS was used to assess the same. In response to the question about social distancing at the vaccination center, an affirmative answer was given by 283 (97.59%) and only three (2.41%) respondents gave negative answers. Many of the vaccine beneficiaries (n = 270, 93.10%) expressed that they were explained about the process of COVID-19 vaccination, and the remaining felt that they were not explained about the process (n = 20, 6.90%). It was observed that the study participants who had expressed that they were informed about the adverse effects were more (n = 264, 91.03%) than the people who felt that they were not informed about the adverse events (n = 26, 8.97%). In the study, 286 (98.62%) respondents reported that they were asked to wait for 30 minutes after the vaccine was administered to them. Whereas only four (1.38%) respondents expressed that they were not made mandatory to wait in the center for 30 minutes after the vaccine was administered to them. The vaccine beneficiaries were instructed to wait for 30 minutes after the COVID-19 vaccination to observe them for immediate side effects, which can be managed at the healthcare facility itself. The number of the study respondents who gave a positive response to the question that asked regarding their overall satisfaction with the vaccination services (n = 280, 96.55%) exceeded far much than those who were not satisfied (n = 10, 3.45%). Satisfaction of the respondents and the influence of their age, gender, and area of residence on the same were studied and are represented in Table 4.

**Table 4:** Influence of age and gender on satisfaction (n = 290). ϰ^2^, chi-square; df, degree of freedom.

Statement		Age (years)			Gender		Area of residence	
		<60		>60	Male	Female	Urban	Rural
Was social distancing maintained at the vaccination site?	Yes (283)	35		248	143	140	244	39
	No (7)	1		6	3	4	7	0
		ϰ^2 ^= 0.02, p = 0.879 at df = 1			ϰ^2 ^= 0.16, p = 0.688 at df = 1		ϰ^2 ^= 1.11, p = 0.291 at df = 1	
Did the staff inform you about the process and give the vaccine properly?	Yes (270)	36	234		136	134	232	38
	No (20)	0	20		10	10	19	1
		ϰ^2 ^= 3.04, p = 0.08 at df = 1			ϰ^2 ^= 0.001, p = 0.97 at df = 1		ϰ^2 ^= 1.31, p = 0.25 at df = 1	
Were you informed about the adverse effects?	Yes (264)	35	229		134	130	227	37
	No (26)	1	25		12	14	24	2
		ϰ^2 ^= 1.92, p = 0.16 at df = 1			ϰ^2 ^= 0.20, p = 0.65 at df = 1		ϰ^2 ^= 0.81, p = 0.36 at df = 1	
Were you asked to wait for 30 minutes post-vaccination for monitoring?	Yes (286)	36	250		145	141	238	38
	No (4)	0	4		1	3	3	1
		ϰ^2 ^= 0.57, p = 0.44 at df = 1			ϰ^2 ^= 1.04, p = 0.30 at df = 1		ϰ^2 ^= 0.41, p = 0.51 at df = 1	
Were you satisfied with the overall experience of vaccination?	Yes (280)	36	244		142	138	244	36
	No (10)	0	10		4	6	7	3
		ϰ^2 ^= 1.46, p = 0.22 at df = 1			ϰ^2 ^= 0.44, p = 0.50 at df = 1		ϰ^2 ^= 2.43, p = 0.11 at df = 1	

As shown in the table, more than 90% of subjects were satisfied for various criteria assessed related to vaccination, but when the subjects were compared as per their age, gender, or area of residence (rural or urban), the difference was not found to be statistically significant.

## Discussion

In the present study, which was conducted in the initial phases of COVID-19 vaccination before the start of the second wave, the majority of the subjects and their family members did not suffer from the COVID-19 infection. Also, the majority had correct knowledge about COVID-19 vaccination and appropriate COVID-19 behavior after vaccination. Fever, body ache, and headache were known to almost one-half and one-third, respectively, of the subjects as adverse effects following immunization. And as expected, social media was the most common source of knowledge. The awareness of the study participants about the COVID-19 vaccine is obvious due to much importance given to the topic and its coverage on a mass scale. However, Mohamed et al. [22], in a web-based study, reported that more than half of their respondents had poor knowledge about the COVID-19 vaccine. This observation on awareness is thus in contrast to that of the present study finding wherein the majority of the respondents were aware of the vaccine. Elgendy and Abdelrahim [23] have also reported that the majority of their participants had good knowledge about the COVID-19 vaccine.

The majority of the subjects were satisfied with the services provided at the vaccination center as assessed by the RAS for satisfaction. The responses in each of the five items in the scale were assessed based on age, gender, and area of residence to identify the influence of these factors on satisfaction. The influence of any of the factors studied, namely, age, gender, or residential status of the subjects, could not be proven statistically significant as the p-value for all the analyses came as more than 0.05. The reason for the same could be the overall satisfaction itself was more and hence no statistically significant difference could be noted in the subgroups. These findings are partly in contrast with those of Malik et al. [5], who conducted a web-based cross-sectional study on vaccine acceptance wherein a gender difference in willingness for the COVID-19 vaccine was reported. Mannan and Farhana [9] also carried out an online survey on vaccine acceptance during the period June-September 2020 in which data were obtained from 26,852 individuals aged more than 19 years on COVID-19 vaccine and observed differences in acceptance of vaccine among various groups. Another researcher also revealed that the study participants were more likely to receive the COVID-19 vaccine if they were younger, females, or more educated [22]. The difference in opinion from the previous studies [2,7,9,22] could be because these studies have assessed the acceptance of the COVID-19 vaccine when it was not available. Whereas the present study has tried to estimate the satisfaction of the vaccine beneficiaries about the vaccine after they have received the shot. Although the acceptance of COVID-19 vaccines has been explicitly surveyed by several other researchers in their studies [24-26], the domain of satisfaction has remained unexplored. Hence, the observations of the present survey on satisfaction of the services at the COVID-19 vaccination center cannot be compared much with other studies since no studies on the topic are available so far.

As awareness about the vaccine will influence its acceptance, the current study will help to detect this aspect. Moreover, it will also serve as a pilot study to assess the overall satisfaction of the study participants about the vaccination services provided at the immunization clinic.

The major strength of the current study is that it is based on face-to-face interviews giving adequate time among participants who voluntarily agreed to consent. This carries importance in the present day scenario with most of the studies being conducted on an online platform. Chances of recall bias are minimal in the study concerning satisfaction as the interviews were conducted immediately after the immunization. Further, the interviewers have encouraged voluntary participation in the study so that compulsion should not result in any response bias.

As with all cross-sectional studies, the current study also has a few limitations, which are inherent to the nature of the study design. Also, the study was carried out on a small sample at a single center where only COVAXIN was administered and at a time when vaccination facilities were available only for the elderly and those with comorbidities. This also explains the inclusion of only the elderly and those with comorbidities as the study participants. Hence, the results of the study can be generalized to individuals with similar socio-demographic characteristics only, which is another limitation of the study. Similar studies on a larger sample with different study designs are recommended to overcome these issues.

## Conclusions

The majority of the study participants in the present survey were aware of the COVID-19 vaccine and adverse events following the administration of COVAXIN. A large majority of the respondents also reported a satisfactory experience at the vaccination center. This indeed is a promising output as the public sector is involved in providing this vaccine. Satisfaction about the public health sector eventually indicates the success of the health department in providing the services to the population and helps in building their trust in the system. Although satisfaction with the vaccination services varied with age, gender, and area of residence, satisfaction levels were overall on a higher side. This satisfaction of the vaccine beneficiaries may act as a positive influence for motivating others for receiving the vaccine. This will lead to enhanced uptake and acceptance of the vaccine by overcoming the existing vaccine hesitancy and skepticism that is widely prevalent in our country.
